# Association Between Pain, Agitation, and Intracranial Pressure with Cerebral Near-Infrared Spectroscopy in Critically Ill Children: A Prospective Pilot Study

**DOI:** 10.3390/jcm15156090

**Published:** 2026-08-05

**Authors:** Fatih Durak, Emine Pinar Kulluoglu, Necati Ozsevil, Berra Bilgin, Gokcen Ozcifci

**Affiliations:** 1 Department of Pediatric Intensive Care Unit, Medical Faculty, Izmir Katip Celebi University, Izmir 35030, Turkey; fatihdurak44@hotmail.com; 2 Department of Pediatric Intensive Care Unit, Izmir City Hospital, Izmir 35030, Turkey; kulluoglupinar@gmail.com (E.P.K.); necati.ozsevil@gmail.com (N.O.); 3 Department of Neurosurgery, Izmir City Hospital, Izmir 35030, Turkey; bilginberra@hotmail.com

**Keywords:** intracranial pressure, near-infrared spectroscopy, cerebral oximetry, neuromonitoring, pediatric intensive care, pain and agitation

## Abstract

**Background/Objectives:** Invasive intracranial pressure (ICP) monitoring is standard in pediatric neurocritical care, but the potential of non-invasive near-infrared spectroscopy (NIRS) and the bedside drivers of ICP remain under-explored. We evaluated the association between cerebral NIRS (rSO_2_), pain and agitation, systemic hemodynamics, inflammatory markers, and invasive ICP in critically ill children. **Methods:** In this prospective pilot study, children undergoing external ventricular-drain-based ICP monitoring were followed with repeated measurements. Linear Mixed Models (LMM) with a random patient intercept were used to assess the NIRS–ICP association, adjusting for hemodynamic (mean arterial pressure (MAP), heart rate) and clinical variables. The Mann–Whitney U test compared parameters across a 20 mmHg ICP threshold, and the Kruskal–Wallis test compared ICP burden across etiology and outcome subgroups. Inflammatory markers (CRP, WBC, procalcitonin) were evaluated separately. **Results:** A total of 596 measurements from 21 patients were analyzed. Cerebral NIRS was the strongest independent predictor of ICP, showing a significant inverse association (estimate −0.609, *p* < 0.001). Agitation was independently associated with higher ICP (estimate 0.856, *p* < 0.001), and this effect was significantly modified by the patient’s behavioral state, being attenuated during sleep and calm wakefulness compared with agitated wakefulness (sleep × agitation interaction, *p* = 0.002). MAP retained an independent positive association with ICP (estimate 0.044, *p* = 0.019), whereas pain score did not. After adjustment for etiology and sex, these associations remained unchanged; etiology was itself an independent predictor of ICP (*p* = 0.001), whereas sex was not. Serum CRP independently predicted ICP (t = 2.382, *p* = 0.018), whereas WBC and procalcitonin did not. ICP burden differed by etiology, with intracranial masses and leukemia showing the highest loads (*p* < 0.001), but not across clinical-outcome groups (*p* = 0.192). **Conclusions:** Cerebral NIRS-derived rSO_2_ was an independent inverse correlate of invasive ICP, and agitation was independently associated with ICP in a state-dependent manner, with its effect attenuated during sleep and calm wakefulness. CRP and etiology further shaped ICP burden. These findings support integrating non-invasive cerebral oximetry into multimodal pediatric neuromonitoring and highlight the value of managing distress to mitigate intracranial hypertension.

## 1. Introduction

Invasive intracranial pressure (ICP) monitoring remains a cornerstone in the management of pediatric patients with acute brain injury, enabling early detection and treatment of intracranial hypertension [1]. The concept of “ICP burden,” reflecting both the magnitude and duration of elevated ICP episodes, has emerged as a key independent predictor of mortality and neurological outcomes [2,3]. In this context, external ventricular drains (EVDs) are considered the gold standard, providing both accurate ICP measurement and therapeutic cerebrospinal fluid drainage [4].

In the pediatric intensive care unit (PICU), common clinical stressors such as pain, agitation, and anxiety may significantly exacerbate intracranial hypertension. These factors can increase ICP through multiple mechanisms, including elevated intrathoracic pressure, impaired cerebral venous return, and increased cerebral metabolic demand [5]. Accordingly, current management strategies emphasize early administration of analgesia and sedation as first-line interventions [1]. However, emerging evidence suggests that commonly used agents, such as fentanyl and midazolam, may have variable and sometimes paradoxical effects on ICP dynamics [6,7].

Given these complexities, there is increasing interest in complementary non-invasive neuromonitoring techniques. Near-infrared spectroscopy (NIRS), which measures regional cerebral oxygen saturation (rSO_2_), has gained attention as a bedside tool for continuous monitoring [8]. Although reductions in rSO_2_ have been associated with elevated ICP, this relationship appears to be heterogeneous and dependent on the underlying pathology [9,10]. Reductions in rSO_2_ have been associated with elevated intracranial pressure and impaired cerebral perfusion, with studies demonstrating improvements in cerebral oxygenation following cerebrospinal fluid diversion in hydrocephalus [11,12]. However, the relationship between rSO_2_ and intracranial dynamics remains complex and influenced by underlying pathology, cerebral blood flow, and metabolic factors. These inconsistencies, together with the limited and heterogeneous pediatric data, highlight the need for further investigation into the relationship between invasive and non-invasive neuromonitoring parameters in critically ill children [13,14,15].

Despite routine clinical management of pain and agitation, their direct and quantifiable effects on intracranial dynamics remain insufficiently characterized in children. Therefore, the aim of this study is to evaluate the relationship between pain and agitation scores (COMFORT-B, FLACC, and Visual Analogue Scale), cerebral NIRS-derived rSO_2_ values, and invasively measured ICP in critically ill pediatric patients. By integrating clinical assessment tools with simultaneous invasive and non-invasive neuromonitoring data, this study seeks to provide insight into how bedside clinical states reflect intracranial physiology and to explore the potential role of NIRS as a complementary monitoring modality in pediatric neurocritical care. We hypothesized that pain and agitation would be associated with ICP, and that cerebral NIRS could serve as a complementary non-invasive marker of intracranial dynamics.

## 2. Materials and Methods

### 2.1. Study Design and Population

This prospective observational cohort study was conducted in a tertiary pediatric intensive care unit (PICU) between January 2026 and July 2026. The study was designed to evaluate the relationship between pain and agitation levels and ICP in critically ill pediatric patients undergoing invasive neuromonitoring. As a pilot study, no formal a priori sample size calculation was performed; all eligible patients admitted during the study period were consecutively enrolled.

Patients aged between 1 month and 18 years who required cerebrospinal fluid (CSF) drainage via an external ventricular drain (EVD) for clinical indications were eligible for inclusion.

Patients were excluded if intracranial pressure monitoring was not available, if clinical instability precluded safe and standardized measurements, or if pain and agitation assessments could not be reliably performed. Patients with insufficient follow-up to obtain at least one complete measurement cycle were excluded to ensure data quality and the validity of the analyses.

### 2.2. Data Collection and Monitoring

Data were collected prospectively during routine clinical monitoring at predefined time points. ICP and NIRS were recorded simultaneously at four fixed time points on each follow-up day (08:00, 12:00, 20:00, and 00:00), performed every three days, supplemented by up to two additional unscheduled measurements per follow-up day obtained during spontaneous clinical events. This protocol yielded 596 paired ICP–NIRS measurements across the cohort, with no missing data for the primary analysis. All ICP, NIRS, pain, and agitation assessments were performed by trained pediatric intensive care physicians following a standardized institutional protocol to ensure consistency across measurements.

Recorded variables included demographic characteristics (age, sex, body weight), underlying diagnosis, date of EVD placement, drainage level, CSF output within the previous 24 h, body temperature prior to measurements, ICP values, pain and agitation scores, clinical status (e.g., asleep, calm, agitated, before or after care procedures), heart rate, non-invasive blood pressure, and respiratory parameters including type of respiratory support and need for mechanical ventilation. The duration of EVD placement and any catheter replacement were recorded. EVD replacement was performed based on clinical indications (e.g., suspected infection or malfunction) rather than routine scheduled exchange.

Additional variables included administration of sedative and analgesic agents and clinical findings such as infection markers, seizures, vomiting, and other neurological signs. Sedative agents were classified as midazolam, dexmedetomidine, or their combination, and analgesic agents as fentanyl or intermittent paracetamol; the requirement for sedation and analgesia at each measurement was recorded as present or absent. Information regarding ICP elevation episodes, need for increased sedation, number of measurements, duration of follow-up, and clinical outcomes was also recorded.

Cerebral oxygenation was continuously monitored using NIRS (INVOS™ 5100C Cerebral/Somatic Oximeter, Medtronic, Minneapolis, MN, USA), which provides non-invasive measurement of regional cerebral oxygen saturation (rSO_2_). Measurements were recorded simultaneously with ICP values. NIRS sensors were placed bilaterally on the frontal region according to the manufacturer’s recommendations. rSO_2_ values were obtained from both hemispheres, and the mean of the right and left measurements was used for analysis.

Data were structured at three hierarchical levels for analysis: individual paired measurements (596 simultaneous ICP–NIRS recordings), patient-day observations (145 patient-days, used for daily aggregated respiratory and clinical parameters), and patient-level summaries (21 patients, aggregated to avoid overrepresentation of repeated measurements in between-group comparisons). The analytic level used for each result is specified in the corresponding table and figure legends.

### 2.3. Intracranial Pressure Measurement

ICP was measured using a fluid-filled transducer system connected to the EVD. During measurements, the drainage system was briefly closed, and ICP values were recorded within 30 s to minimize the impact on intracranial dynamics. This method represents a well-established and validated approach for intracranial pressure monitoring [16].

Invasive ICP monitoring via ventricular catheters is widely accepted as the gold standard method for intracranial pressure measurement due to its accuracy and reliability, as well as its ability to provide both monitoring and therapeutic CSF drainage [17].

### 2.4. Pain and Agitation Assessment

Pain and agitation levels were assessed simultaneously with ICP measurements using validated pediatric scales. The COMFORT-Behavior (COMFORT-B) scale and the Face, Legs, Activity, Cry, Consolability (FLACC) scale were used for behavioral assessment, while the Visual Analogue Scale (VAS) was applied when appropriate based on the patient’s age and clinical condition [18,19,20].

### 2.5. Statistical Analysis

Continuous variables were expressed as median (interquartile range, IQR 25th–75th percentile), as most variables were non-normally distributed; normality was assessed using the Shapiro–Wilk test. Categorical variables were compared using the Pearson chi-square test (or Fisher’s exact test where expected cell counts were low), and adjusted standardized residuals were examined to identify categories driving significant associations. Between-group comparisons of continuous variables according to the clinically significant ICP threshold of 20 mmHg were performed using the Mann–Whitney U test. The relationship between NIRS and ICP was first evaluated using Spearman’s correlation, then modeled using Linear Mixed Models (LMM) with a random patient intercept to account for repeated longitudinal measurements, adjusting for hemodynamic confounders (MAP, heart rate), behavioral and clinical status, etiology, and sex. Interaction terms between behavioral state and pain/agitation scores were specified a priori on clinical grounds, and the model including these terms was retained after confirming a lower Akaike Information Criterion relative to a main-effects-only model. Denominator degrees of freedom were estimated using the Satterthwaite approximation, as implemented by default in the SPSS MIXED procedure (version 23.0). Multicollinearity was assessed using the variance inflation factor (VIF) in all multivariable models. In the primary model, age-standardized hemodynamic measures were excluded to avoid redundancy with their raw counterparts; thereafter all VIF values were below 5 in both the primary and the inflammatory-marker models (range 1.01–3.44), indicating no significant multicollinearity. Inflammatory markers and clinical risk factors were incorporated into separate multivariable mixed models. Differences across etiology, diagnosis, and clinical-outcome groups were analyzed using the Kruskal–Wallis test after aggregating data to the patient level to avoid overrepresentation of repeated observations. A two-sided *p* < 0.05 was considered statistically significant. Statistical analyses were performed using SPSS (version 23.0, IBM Corp., Armonk, NY, USA). No data were missing for the primary mixed-model analysis; all 596 paired measurements were included.

## 3. Results

The study included a total of 21 patients. The demographic and clinical characteristics of the study cohort at baseline are summarized in Table 1. The study population comprised 15 males (71.4%) and 6 females (28.6%). The median age of the participants was 17 months (interquartile range (IQR) 25–75p: 6.5–100 months), and the median body weight was 10 kg (IQR 25–75p: 8–25 kg). Regarding the primary diagnosis, congenital hydrocephalus was the most prevalent condition, identified in 9 (42.9%) of the cases, followed by intracranial masses in 6 (28.6%) patients. Clinical indications for EVD included shunt infection in 10 (47.6%) patients, new-onset hydrocephalus in 6 (28.6%) patients, and shunt dysfunction in 5 (23.8%) patients.

Baseline clinical parameters and laboratory markers were recorded upon admission. The median baseline intracranial pressure (ICP) was 10.75 mmHg (IQR 25–75p: 7.5–18.75 mmHg), while the median regional cerebral oxygen saturation (NIRS) was 83% (IQR 25–75p: 72.5–88%). Laboratory findings revealed a median C-reactive protein (CRP) level of 24 mg/L (IQR 25–75p: 7–78 mg/L) and a median white blood cell (WBC) count of 11,160/µL (IQR 25–75p: 7950–15,455/µL).

Throughout the follow-up period, the median daily EVD drainage volume was 8.45 mL/kg/day (IQR 25–75p: 2.55–16.1 mL/kg/day). Among the total observations, 269 (30.9%) were performed during sleep, and 374 (43%) were conducted before routine care. Supplementary sedation was required in 23 measurements, and supplementary analgesia was administered in 36. Across 596 total measurements, the mean heart rate standard deviation score (SDS) was 0.74 ± 0.072, and the mean arterial pressure SDS was 0.595 ± 0.055.

**Figure 1:**
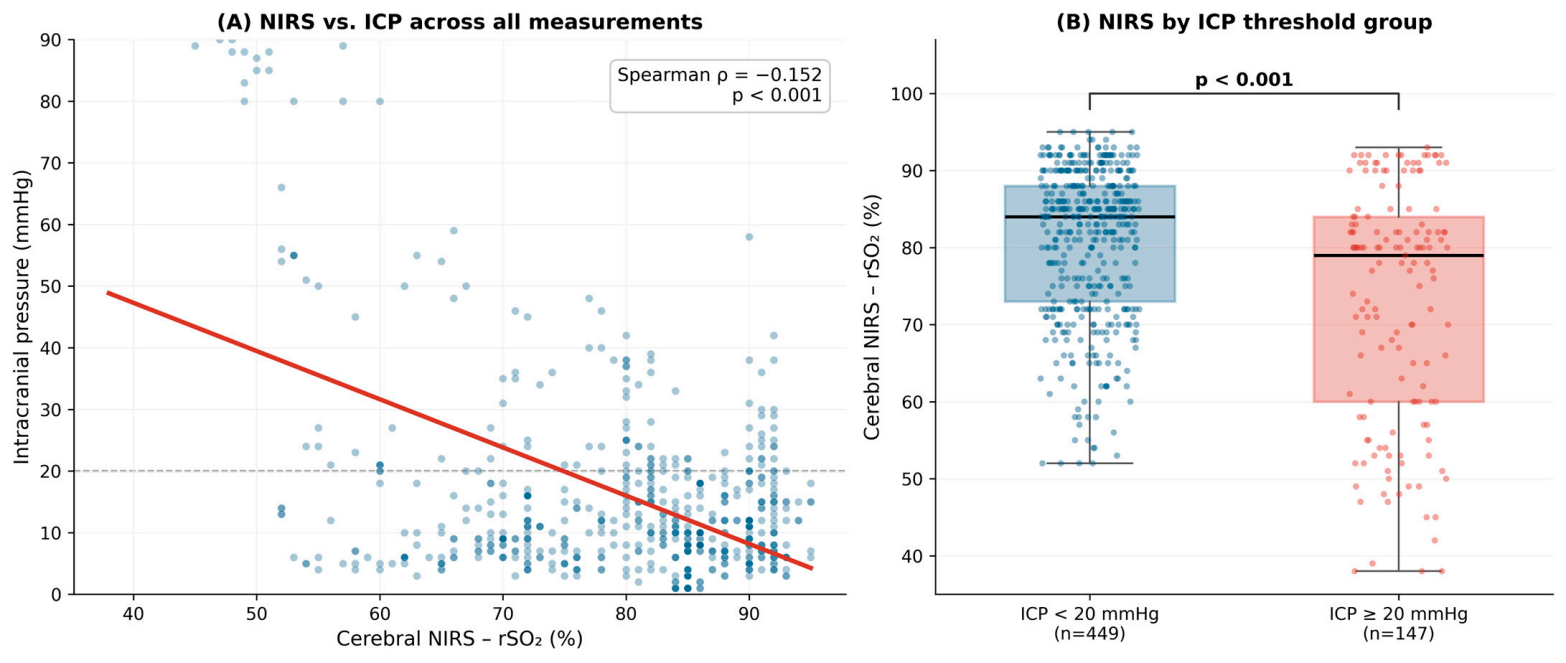
Association between cerebral regional oxygen saturation (NIRS, rSO_2_) and invasively measured intracranial pressure (ICP). (**A**) Scatter of all paired measurements (*n* = 596); higher rSO_2_ was associated with lower ICP (Spearman’s ρ = −0.152, *p* < 0.001), and the dashed line marks the 20 mmHg ICP threshold. (**B**) NIRS values in measurements with ICP < 20 mmHg (median 84%, *n* = 449) versus ICP ≥ 20 mmHg (median 79%, *n* = 147); rSO_2_ was significantly lower in the high-ICP group (Mann–Whitney U test, *p* < 0.001). Boxes show median and interquartile range; whiskers extend to 1.5× IQR.

Initial univariate correlation analysis revealed significant positive associations between ICP and pain scores (ρ = 0.286, *p* < 0.001) as well as agitation scores (ρ = 0.169, *p* < 0.001), while NIRS showed a significant inverse correlation with ICP (ρ = −0.152, *p* < 0.001) (Figure 1A). However, in the subsequent linear mixed model adjusting for systemic hemodynamic variables (MAP, heart rate) and clinical status, the inverse association between NIRS and ICP remained highly significant, while agitation retained an independent positive association with ICP and pain did not. Notably, the effect of agitation on ICP was dependent on the patient’s behavioral state, being attenuated during sleep and calm wakefulness (detailed below), indicating that both the intensity of agitation and the underlying patient state shape intracranial pressure.

Patients were categorized based on a clinically significant ICP threshold of 20 mmHg to determine predictive clinical factors. Comparative analysis using the Mann–Whitney U test demonstrated that patients with ICP ≥ 20 mmHg exhibited significantly higher pain scores (*p* < 0.001) and MAP values (*p* < 0.001) compared to those with ICP < 20 mmHg. Heart rate SDS was also significantly higher in the high-ICP group (*p* < 0.001). Furthermore, NIRS values were significantly lower in the high-ICP cohort (*p* < 0.001) (Figure 1B). These findings indicate that elevated pain levels and systemic hypertension are strongly associated with critical intracranial pressure thresholds, and that NIRS serves as a robust monitoring tool for identifying patients at risk of intracranial hypertension.

A linear mixed model was employed to evaluate the factors influencing intracranial pressure over time, accounting for individual patient variability as a random effect (Table 2). To avoid redundancy between raw and age-standardized hemodynamic measures, only raw mean arterial pressure and heart rate were retained. The final model included interaction terms between behavioral state and pain/agitation, specified a priori on clinical grounds; this model was preferred over a main-effects-only model based on a lower Akaike Information Criterion (3728.5 vs. 3779.4). In this model, regional cerebral oxygen saturation was the most significant independent predictor of ICP (estimate: −0.609, 95% CI: −0.700 to −0.519, *p* < 0.001). This inverse relationship suggests a bidirectional dynamic: high ICP may compromise cerebral perfusion, while effective ICP management is associated with improved NIRS values, positioning NIRS as a reliable surrogate marker for intracranial stability. MAP also showed a significant positive association with ICP (estimate: 0.044, 95% CI: 0.007 to 0.080, *p* = 0.019), underscoring the impact of systemic blood pressure fluctuations on intracranial hemodynamics. The fixed effects explained 34% of the variance in ICP (marginal R^2^ = 0.34), while the full model including patient-level random effects explained 96% (conditional R^2^ = 0.96); the intraclass correlation of 0.94 indicates that the large majority of ICP variability was attributable to between-patient differences.

**Figure 2:**
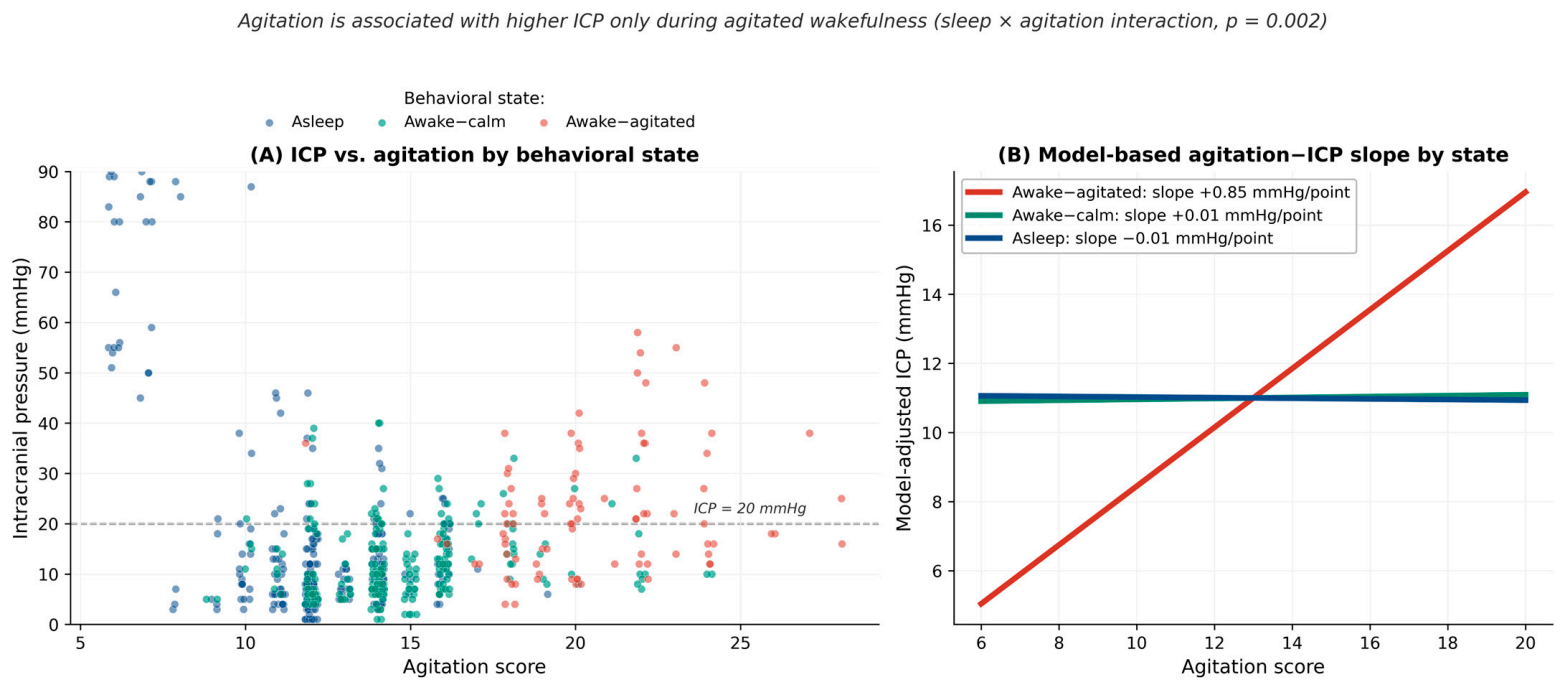
Relationship between agitation score and ICP stratified by behavioral state. (**A**) Raw paired measurements colored by behavioral state (asleep, awake-calm, awake-agitated); the dashed line marks the 20 mmHg ICP threshold. (**B**) Model-based agitation–ICP slopes from the linear mixed model: agitation was associated with higher ICP only during agitated wakefulness (slope +0.85 mmHg per point), with the effect effectively abolished during sleep and calm wakefulness (sleep × agitation interaction, *p* = 0.002).

Regarding patient state, agitation score was independently and positively associated with ICP (estimate: 0.856, *p* < 0.001), whereas pain score showed no independent association in the adjusted model (*p* = 0.448). Importantly, a significant interaction was observed between sleep status and agitation (*p* = 0.002): the ICP-elevating effect of agitation was greatest during agitated wakefulness and was significantly attenuated both during sleep (interaction estimate: −0.873, *p* = 0.002) and during calm wakefulness (interaction estimate: −0.842, *p* = 0.001). In other words, the same level of agitation was associated with a markedly smaller rise in ICP when the patient was asleep or calm than when agitated and awake (Figure 2). No significant interactions were found between sleep status and pain, or between care status and either pain or agitation (all *p* > 0.13). These findings indicate that both the intensity of agitation and the underlying behavioral state independently shape intracranial pressure, suggesting that maintaining a calm patient state may attenuate agitation-driven ICP elevations in pediatric neurocritical care.

To further explore clinical drivers of intracranial hypertension, we examined the association between various clinical interventions/complications and the ICP threshold of 20 mmHg (Table 3). Crosstabulation analysis revealed that the requirement for continuous sedation (*p* = 0.001) and analgesia (*p* < 0.001), the presence of infection signs (*p* = 0.001), and new-onset seizures (*p* = 0.003) were significantly more frequent in the high-ICP group, whereas vomiting did not differ significantly between groups (*p* = 0.158). The distribution of respiratory support also differed significantly (*p* < 0.001), with invasive mechanical ventilation markedly overrepresented in the high-ICP group. These findings suggest that patients requiring intensive pharmacological management and those displaying signs of systemic infection are at a substantially higher risk of developing intracranial hypertension.

To elucidate the relationship between systemic inflammation and intracranial hypertension, we incorporated inflammatory markers (CRP, WBC, PCT) into a separate linear mixed model (Table 4). The analysis identified serum CRP as a statistically significant predictor of ICP (estimate 0.052, t = 2.382, *p* = 0.018), suggesting that systemic inflammatory states directly correlate with intracranial pressure instability. Conversely, while WBC and PCT levels were monitored, they did not reach independent statistical significance in this multivariable model. All variance inflation factors in this model were below 1.2, confirming the absence of multicollinearity. These findings underscore the critical role of systemic inflammation, specifically CRP-mediated pathways, in the pathophysiology of intracranial hypertension in this cohort.

Kruskal–Wallis analysis demonstrated that intracranial pressure dynamics varied significantly according to the underlying etiology (*p* < 0.001 for daily mean ICP) (Table 5). Patients diagnosed with intracranial tumors or leukemia exhibited higher cumulative ICP loads compared to congenital cases. When patients were stratified by underlying diagnostic category, daily mean ICP did not differ significantly (*p* = 0.552), although the ICP rise-episode rate did differ across these groups (*p* = 0.018). Conversely, when patients were stratified by clinical outcomes (e.g., shunt placement, EVD revision, stable status), no statistically significant difference was observed in daily mean ICP values (*p* = 0.192). These findings indicate that while etiology is a strong determinant of ICP intensity, the clinical decision for definitive surgical intervention (such as shunt placement) is likely influenced by multi-parametric clinical indicators beyond ICP thresholds alone.

## 4. Discussion

In this prospective pilot study of critically ill children undergoing EVD-based invasive ICP monitoring, we simultaneously integrated bedside clinical assessments (pain and agitation scores), non-invasive cerebral NIRS, systemic hemodynamic parameters, and inflammatory markers to characterize the physiological determinants of intracranial pressure. Our principal findings were fourfold: (1) cerebral rSO_2_ was the strongest independent inverse correlate of ICP; (2) agitation was independently associated with ICP, with its effect significantly modified by the patient’s behavioral state (sleep versus agitated wakefulness), while systemic MAP remained an independent hemodynamic contributor; (3) systemic inflammation, reflected by serum CRP, was independently associated with ICP; and (4) ICP burden differed markedly by underlying etiology but not by ultimate clinical disposition.

In our linear mixed model, regional cerebral oxygen saturation emerged as the most significant independent predictor of ICP, with higher rSO_2_ values corresponding to lower ICP. This inverse relationship is physiologically coherent: sustained intracranial hypertension compromises cerebral perfusion pressure and, consequently, cerebral oxygen delivery, which NIRS detects at the bedside as intracranial hypertension can lead to cerebral ischemia and brain tissue hypoxia [11]. Our findings align with perioperative data in symptomatic pediatric hydrocephalus, in which rSO_2_ improved following cerebrospinal fluid diversion, supporting the notion that effective ICP reduction is accompanied by measurable gains in cerebral oxygenation [11]. Nonetheless, the rSO_2_–ICP relationship is not universally linear; prior work has emphasized that it is modulated by underlying pathology, cerebral blood flow, PaCO_2_, and hemoglobin concentration [21]. A recent pediatric study combining NIRS with transcranial Doppler and optic nerve sheath measurements similarly concluded that no single non-invasive modality perfectly captures elevated ICP, arguing for a multimodal approach [22]. Our data extend these observations by quantifying, within a repeated-measures framework, the magnitude of the rSO_2_–ICP association while adjusting for hemodynamic confounders, and they support the use of NIRS as a complementary—though not stand-alone—surrogate marker of intracranial stability in children with abnormal cerebral anatomy such as hydrocephalus and shunt malfunction [23,24].

In our mixed model, agitation was independently and positively associated with ICP, and—unlike in a simpler additive model—this effect was significantly modified by the patient’s behavioral state, as reflected by a significant sleep × agitation interaction. The ICP-elevating influence of agitation was greatest during agitated wakefulness and was substantially attenuated both during sleep and during calm wakefulness. This finding is physiologically coherent: agitation and ventilator dyssynchrony raise intrathoracic pressure, impair cerebral venous return, and increase systemic arterial pressure, which in turn can elevate ICP in patients operating at the steep portion of their cerebral autoregulatory curve [25,26]. Mean arterial pressure retained an independent positive association with ICP in our model, consistent with this hemodynamic pathway. The observation that the same level of agitation translates into a smaller rise in ICP when the child is asleep or calm provides direct, quantitative support for the “tier-zero” recommendation that analgesia and sedation be optimized in all neurocritical care patients to prevent distress-mediated ICP surges [25]. This state-dependent pattern also echoes observations that under-sedation and autonomic instability magnify ICP [27]. Notably, pain score was not independently associated with ICP once agitation and hemodynamics were accounted for, suggesting that in this cohort the behavioral manifestation of distress—agitation—rather than nociception per se was the more proximate driver of intracranial pressure, although the paradoxical and agent-dependent effects of fentanyl and midazolam on ICP warrant caution and further study [28,29]. Collectively, these results reframe pain and agitation not as uniform, additive contributors to ICP but as state-dependent influences, reinforcing the clinical value of maintaining a calm patient state in pediatric neurocritical care.

Incorporating inflammatory markers into a separate mixed model, we identified serum CRP as an independent predictor of ICP, whereas WBC and procalcitonin did not reach significance. This is biologically plausible in an EVD cohort, in which nearly half of our patients underwent drainage for shunt infection: ventriculitis and intracranial inflammation impair CSF reabsorption at the arachnoid villi, promoting hydrocephalus and ICP elevation [30,31]. EVDs themselves act as conduits for infection and amplify local inflammatory signaling, linking systemic and intracranial inflammatory states [32]. Elevated CRP has been associated with in-hospital neurological deterioration after acute brain injury, consistent with a broader role for systemic inflammation in secondary brain injury [33]. Our finding that CRP, rather than WBC or PCT, was the discriminating marker may reflect the sensitivity of CRP to low-grade or subclinical inflammation not yet manifest as leukocytosis, though this observation should be interpreted cautiously given the modest sample size.

ICP burden varied significantly by admission etiology, with children harboring intracranial masses or leukemic CNS involvement demonstrating the highest cumulative ICP loads, and neonatal intraventricular hemorrhage sequelae and head trauma the lowest. This gradient is consistent with the concept that space-occupying and infiltrative pathologies more consistently breach compensatory reserve than post-hemorrhagic or dysfunctional-shunt states. In contrast, when patients were stratified by underlying diagnostic category, daily mean ICP did not differ significantly, although the rate of ICP-elevation episodes did—suggesting that intermittent surges, rather than sustained baseline elevation, may better distinguish diagnostic groups. Notably, ICP metrics did not differ across ultimate clinical outcomes (stable status, conversion to shunt, EVD revision, death). This dissociation reinforces a central tenet of contemporary neurocritical care: the decision for definitive surgical intervention is multi-parametric, integrating clinical trajectory, imaging, and CSF dynamics rather than ICP thresholds in isolation [16]. It also echoes evidence from pediatric traumatic brain injury that both the magnitude and duration of intracranial hypertension—the “ICP burden”—rather than any single value, drive outcome [34]. 

The high conditional R^2^ (0.96) contrasted with a modest marginal R^2^ (0.34), and the intraclass correlation of 0.94 demonstrated that most of the variability in ICP was explained by stable between-patient differences rather than by the measured time-varying covariates. This substantial individual heterogeneity suggests that ICP behavior in critically ill children is highly patient-specific, and it underscores that population-level fixed-effect estimates should be applied cautiously to individual patients—reinforcing the value of individualized, multi-parameter neuromonitoring over reliance on group-average thresholds.

## 5. Limitations

This study has several limitations. First, the sample size was small (21 patients), and the single-center design limits generalizability; the analysis should be regarded as a hypothesis-generating pilot. Second, although the linear mixed model incorporated numerous covariates, the ratio of predictors to patients raises the possibility of overfitting, and the model estimates should be interpreted with corresponding caution. Third, several subgroups in the Kruskal–Wallis analyses (e.g., death, stable-without-EVD) contained very few patients, limiting statistical power and precluding robust between-group inference for these categories. Fourth, the observational design precludes causal inference; the temporal and directional relationships between agitation, systemic hemodynamics, and ICP—including the state-dependent modulation we observed—require confirmation in prospective, time-resolved analyses. Fifth, NIRS reflects regional rather than global cerebral oxygenation and is influenced by extracranial contamination, probe position, and abnormal cerebral anatomy, all of which may attenuate its correlation with ICP. Finally, ICP-related outcomes were derived from aggregated per-patient data, which, while appropriate to avoid overrepresentation of repeated measurements, reduces the granularity of the temporal ICP signal.

## 6. Conclusions

In critically ill children with EVDs, cerebral NIRS-derived rSO_2_ was an independent inverse correlate of invasively measured ICP, and agitation was independently associated with ICP in a state-dependent manner, with its effect attenuated during sleep and calm wakefulness. Systemic inflammation, reflected by CRP, and underlying etiology further shaped ICP burden. Together, these findings support the integration of non-invasive cerebral oximetry into multimodal neuromonitoring and highlight the value of managing distress and hemodynamics to mitigate intracranial hypertension. Larger, prospective, time-resolved studies are needed to confirm these state-dependent associations and to define the role of NIRS as a complementary monitoring modality in pediatric neurocritical care.

## Figures and Tables

**Table 1:** Baseline demographic, clinical, and laboratory characteristics of the study cohort (21 patients; 596 paired ICP–NIRS measurements across 145 patient-days).

Characteristic	Value
Sex, male	15 (71.4%)
Age, months *	17.0 (6.5–100.0)
Body weight, kg *	10.0 (8.0–25.0)
Primary Diagnosis	
Congenital hydrocephalus	9 (42.9%)
Intracranial mass	6 (28.6%)
Neonatal IVH sequela	3 (14.3%)
Other (Trauma, Leukemia, AVM)	3 (14.3%)
Indication for EVD	
Shunt infection	10 (47.6%)
New-onset hydrocephalus	6 (28.6%)
Shunt dysfunction	5 (23.8%)
Baseline Parameters * (patient-day level, *n* = 145)	
Baseline ICP, mmHg	10.75 (7.5–18.75)
Baseline NIRS (rSO_2_), %	83 (72.5–88)
CRP, mg/L	24 (7–78)
WBC, /µL	11,160 (7950–15,455)
Daily EVD drainage, mL/kg/day	8.45 (2.55–16.1)
Clinical Management	
Sedation administered, *n* (%) of patients	10 (47.6%)
—Midazolam	4 (40.0%)
—Dexmedetomidine	5 (50.0%)
—Midazolam + dexmedetomidine	1 (10.0%)
Analgesia administered, *n* (%) of patients	13 (61.9%)
—Fentanyl	6 (46.2%)
—Intermittent paracetamol	7 (53.8%)
Measured during sleep	269 (30.9%)
Measured before routine care	374 (43.0%)
Follow-up duration, days *	19 (16–22)
Respiratory Support (patient-day level, *n* = 145)	
None	100 (69%)
Supplemental oxygen	12 (8.3%)
High-flow nasal cannula oxygen therapy	4 (2.8%)
Non-invasive ventilation	2 (1.4%)
Invasive mechanical ventilation	27 (18.6%)

* Values are expressed as median (interquartile range). IQR = interquartile range (25th–75th percentile); EVD = external ventricular drain; CRP = C-reactive protein; WBC = white blood cell count.

**Table 2:** Linear mixed model for factors associated with intracranial pressure (ICP) over time (dependent variable: ICP; random intercept by patient; *n* = 596 measurements, 21 patients).

Predictor	Estimate	SE	df	t	95% CI	*p*
Primary Predictors						
NIRS (rSO_2_)	**−0.609**	**0.046**	**568.9**	**−13.167**	**−0.700, −0.519**	**<0.001**
MAP	**0.044**	**0.019**	**441.8**	**2.346**	**0.007, 0.080**	**0.019**
Agitation score	**0.856**	**0.227**	**439.5**	**3.776**	**0.411, 1.302**	**<0.001**
Pain score	−0.272	0.357	441.6	−0.760	−0.974, 0.431	0.448
Behavioral State (ref: awake-agitated)						
Sleep status: asleep	**10.457**	**4.375**	**443.7**	**2.390**	**1.859, 19.055**	**0.017**
Sleep status: awake-calm	**10.972**	**4.184**	**435.3**	**2.623**	**2.749, 19.195**	**0.009**
Etiology (ref: AV malformation)						
Head trauma	2.181	7.988	130.7	0.273	−13.622, 17.983	0.785
Congenital	**12.437**	**6.123**	**131.9**	**2.031**	**0.325, 24.549**	**0.044**
Neonatal IVH	5.077	6.803	130.8	0.746	−8.382, 18.536	0.457
Intracranial mass	**19.897**	**6.079**	**130.8**	**3.273**	**7.871, 31.922**	**0.001**
Leukemia	**15.557**	**6.853**	**132.3**	**2.270**	**2.002, 29.112**	**0.025**
Etiology (overall, Type III)	**—**	**—**	**5131.9**	**F = 4.625**	**—**	**0.001**
Interaction Terms						
Sleep(asleep) × agitation	**−0.873**	**0.280**	**451.0**	**−3.120**	**−1.422, −0.323**	**0.002**
Sleep(awake-calm) × agitation	**−0.842**	**0.247**	**437.8**	**−3.402**	**−1.328, −0.355**	**0.001**
Adjusted Covariates (all non-significant)						
Sex (male ref.), heart rate, care status, sedation, analgesia	—	—	—	—	—	0.193–0.948
Random effects (Wald Z)						
Residual variance	11.391	0.779	—	14.615	9.961, 13.025	<0.001
Intercept variance (patient)	187.834	23.681	—	7.932	146.709, 240.486	<0.001

SE = standard error; df = Satterthwaite-approximated denominator degrees of freedom; CI = confidence interval. Bold values indicate statistically significant fixed effects (*p* < 0.05). Non-significant interaction terms (sleep × pain, care × pain, care × agitation; all *p* > 0.18) were included in the model but are not shown. Etiology and sex were included as covariates; etiology was a significant predictor overall (*p* = 0.001), whereas sex was not (*p* = 0.193). The model was also adjusted for follow-up day and time-of-day (both non-significant).

**Table 3:** Comparison of pain, agitation, and hemodynamic/NIRS parameters between measurements with ICP ≥ 20 mmHg and ICP < 20 mmHg (independent-samples Mann–Whitney U test; measurement-level data, *n* = 596).

Variable	ICP < 20 mmHg (*n* = 449)	ICP ≥ 20 mmHg (*n* = 147)	*p*	
Pain score	**0.0 (0.0–1.0)**	**1.0 (0.0–3.0)**	**<0.001**	
Agitation score	14.0 (12.0–16.0)	14.0 (10.0–20.0)	0.117	
Heart rate	116.0 (101.0–132.0)	119.0 (97.0–140.0)	0.349	
Heart rate SDS	**0.42 (−0.73–1.54)**	**1.33 (−0.07–2.83)**	**<0.001**	
MAP	**74.0 (67.0–82.0)**	**81.0 (73.0–90.0)**	**<0.001**	
MAP SDS	0.40 (−0.26–1.26)	0.60 (−0.20–1.40)	0.257	
NIRS (rSO_2_)	**84.0 (73.0–88.0)**	**79.0 (60.0–84.0)**	**<0.001**	
Clinical parameter, *n* (%)	**ICP < 20 mmHg**	**ICP ≥ 20 mmHg**	***p*** **value and** **Adj. residual (ICP ≥ 20)**	
Sedation required	**125 (27.8%)**	**62 (42.2%)**	**0.001**	**+3.3**
Analgesia required	**105 (23.4%)**	**56 (38.1%)**	**<0.001**	**+3.5**
Infection signs present	**58 (12.9%)**	**36 (24.5%)**	**0.001**	**+3.3**
New seizure onset	**31 (6.9%)**	**22 (15.0%)**	**0.003**	**+3.0**
Vomiting	25 (5.6%)	13 (8.8%)	0.158	+1.4
Respiratory support				
None	**353 (78.6%)**	**59 (40.1%)**	**<0.001**	**−8.8**
Supplemental oxygen	**29 (6.5%)**	**21 (14.3%)**		**+3.0**
High-flow nasal cannula	10 (2.2%)	7 (4.8%)		+1.6
Non-invasive ventilation	8 (1.8%)	0 (0.0%)		−1.6
Invasive mechanical ventilation	**49 (10.9%)**	**60 (40.8%)**		**+8.1**

Values are *n* (%) within each ICP group. The adjusted (standardized) residual is shown for the ICP ≥ 20 mmHg group; values exceeding ±2 indicate cells contributing significantly to the association (positive = observed higher than expected under independence). In 2 × 2 comparisons the residual for the ICP < 20 group is equal in magnitude and opposite in sign. Bold rows denote statistically significant group differences (*p* < 0.05); for respiratory support the overall distribution differed between groups (*p* < 0.001), with the high-ICP group showing fewer measurements on no support and more on supplemental oxygen and invasive mechanical ventilation. Fever was constant (absent in all measurements) and is not shown.

**Table 4:** Separate linear mixed model incorporating inflammatory markers as predictors of ICP.

Predictor	Estimate	SE	df	t	95% CI	*p*
CRP	**0.052**	**0.022**	**167.784**	**2.382**	**0.009, 0.096**	**0.018**
PCT	0.345	0.206	113.005	1.674	−0.063, 0.753	0.097
WBC (blood)	<0.001	<0.001	490.673	−0.106	−0.000316, 0.000284	0.916
WBC (CSF)	<0.001	0.001	113.074	0.395	−0.001, 0.002	0.694
CSF culture growth (ref: no growth)	0.869	6.226	116.212	0.139	−11.463, 13.200	0.889

SE = standard error; df = Satterthwaite-approximated denominator degrees of freedom; CI = confidence interval; CRP = C-reactive protein; PCT = procalcitonin; WBC = white blood cell; CSF = cerebrospinal fluid. Bold values indicate statistically significant predictors (*p* < 0.05). The model was adjusted for a random patient intercept.

**Table 5:** Kruskal–Wallis comparison of ICP burden across etiology, diagnosis, and clinical-outcome subgroups.

Grouping Variable/Subgroup	*n*	Daily Mean ICP, Median (IQR 25–75)	Elevated ICP Rate (≥20 mmHg), Median (IQR)
Etiology			
Leukemia	13	17.0 (10.9–22.1)	0.25 (0.00–0.63)
Intracranial mass	38	13.3 (8.7–34.3)	0.00 (0.00–0.81)
Congenital	62	13.6 (7.5–19.9)	0.00 (0.00–0.75)
AV malformation	6	9.6 (9.1–10.1)	0.00 (constant)
Head trauma	6	8.3 (6.3–9.0)	0.00 (constant)
Neonatal IVH	20	6.8 (4.8–9.2)	0.00 (0.00–0.00)
Overall (ICP)	145	***p*** **< 0.001**	
Overall (elevated ICP rate)	145	***p*** **= 0.002**	
Underlying diagnosis			
Shunt infection	71	11.3 (6.5–21.8)	0.00 (0.00–0.75)
Shunt dysfunction	36	11.3 (7.2–18.8)	0.00 (0.00–0.50)
New hydrocephalus	38	9.6 (7.8–14.1)	0.00 (0.00–0.00)
Overall (ICP)	145	*p* = 0.552	
Overall (elevated ICP rate)	145	***p*** **= 0.018**	
Clinical outcome			
Death	2	31.4 (9.0–53.8)	0.50 (0.00–1.00)
EVD revision	10	16.8 (10.0–22.8)	0.29 (0.00–0.75)
Stable (without EVD)	1	12.3 (constant)	0.00 (constant)
Stable (with EVD)	122	10.8 (7.3–18.8)	0.00 (0.00–0.50)
Converted to shunt	10	9.4 (5.5–11.4)	0.00 (constant)
Overall (ICP)	145	*p* = 0.192	
Overall (elevated ICP rate)	145	*p* = 0.079	

Values are medians (interquartile range, IQR 25th–75th percentile) of patient-day values within each subgroup (*n* = 145 patient-days across 21 patients). “Daily mean ICP” (mmHg) is the mean of each day’s ICP measurements; “elevated ICP rate” (0–1) is the fraction of each day’s measurements with ICP ≥ 20 mmHg. Subgroups are ordered from highest to lowest daily mean ICP. “Overall” rows give the Kruskal–Wallis *p*-value for each outcome; bold denotes *p* < 0.05. “Constant” indicates no variation (single patient or identical values), so no IQR is defined. Some subgroups were very small (death, *n* = 2; stable without EVD, *n* = 1) and are underpowered; comparisons involving these should be interpreted with caution.

## Data Availability

The data presented in this study are available on request from the corresponding author due to privacy restrictions relating to pediatric patient data.
